# Refinement of maximal levator resection for blepharoptosis correction: High incision and advancement of levator complex

**DOI:** 10.1016/j.jpra.2026.02.004

**Published:** 2026-02-10

**Authors:** Yu-Chi Wang, Chung-Sheng Lai

**Affiliations:** Division of Plastic Surgery, Department of Surgery, Kaohsiung Medical University Hospital, Kaohsiung, Taiwan

**Keywords:** Blepharoptosis, Conjunctival perforation, High levator incision, Maximal levator resection

## Abstract

**Background:**

Maximal levator resection (MLR) is indicated for severe blepharoptosis with poor levator function (LF) but carries risks of intraoperative bleeding due to dissection above the tarsus, conjunctival perforation, and postoperative conjunctival prolapse. To address these issues, we introduce the High Incision and Advancement of Levator Complex (HIAL) technique, designed to optimizing functional and aesthetics outcomes while minimizing complications.

**Objectives:**

This study evaluates the effectiveness of HIAL technique in reducing surgical complications in severe congenital blepharoptosis.

**Methods:**

Nine pediatric patients with congenital severe blepharoptosis underwent HIAL procedure. The levator complex was dissected 8–10 mm above the superior tarsal border and extended toward Whitnall’s ligament to create a 10 × 5 mm rectangular flap, which was then advanced and secured to the tarsus.

**Results:**

The mean levator resection measured 24.67 ± 0.87 mm. Postoperative outcomes demonstrated significant improvements in palpebral fissure height (PFH), marginal reflex distance 1 (MRD1), and LF (all *p* < 0.05). Functionally, seven patients achieved excellent outcomes, and two demonstrated good results, with all exhibiting favorable aesthetics and no recurrence.

**Conclusions:**

The HIAL technique may represent a feasible and potentially effective option for managing severe blepharoptosis with poor LF, providing both functional and aesthetic benefits while reducing surgical complications.

## Introduction

Severe congenital blepharoptosis is characterized by eyelid drooping exceeding 4 mm and poor levator function, which can lead to amblyopia and impaired visual development in pediatric patients.^1^ MLR was developed as an alternative approach to address these limitations.^3^ However, MLR presents challenges, including potential injury to the marginal and peripheral vascular arcades during deep dissection, which increases the risk of intraoperative bleeding. Furthermore, persistent excess conjunctival tissue following resection may result in postoperative conjunctival prolapse.^2^^,^^3^

To address these concerns, we developed the HIAL technique. This approach involves dissecting a rectangular levator flap 8–10 mm above the superior tarsal border and advancing it to the tarsus, aiming to reduce complications while achieving improved functional and aesthetic outcomes.

## Patients and methods

A retrospective case series study was conducted at a single medical center between January 2018 and June 2023, following institutional review board approval and in compliance with the Declaration of Helsinki.

### Surgical technique

The surgical procedure of HIAL was demonstrated in Video 1 and supplementary information (SI) 1A-D.

1. Surgery was performed under general anesthesia with the patient in the supine position.
2. Dissection of the levator complex proceeded superiorly toward Whitnall’s ligament (SI 1A). A high incision line was marked approximately 8–10 mm above the superior tarsal border, guided by the patient’s central tarsal height. A rectangular advancement flap measuring approximately 10 mm in vertical height and 5 mm in horizontal width was then designed (Video 1, SI 1B).
3. The flap was incised along the previously marked boundaries through Muller’s muscle, carefully separated from the underlying conjunctiva, and dissected superiorly beyond Whitnall’s ligament (Video 1, SI 1C).
4. The flap was anchored to the midpoint and upper one-third of the tarsus using 6–0 nylon suture (SI 1D). Final refinement of eyelid margin position was achieved by aligning the margin with the superior limbus and placing additional medial and lateral sutures as needed (Video 1).
5. The upper eyelid crease was created by anchoring the subdermal tissue to the pretarsal levator aponeurosis with 6–0 nylon sutures.

## Results

A total of nine patients with unilateral severe blepharoptosis were included in the study (Table 1). The mean preoperative LF was 3.00 ± 1.12 mm. The mean total advancement of the levator complex, which included both the length of flap advancement and the distance of high incision, was 24.67 ± 0.87 mm. Postoperatively, PFH (2.00 ± 1.32 mm vs. 8.11 ± 0.60 mm, *p* < 0.05), MRD1 (−3.00 ± 1.32 mm vs. 3.22 ± 0.67 mm, *p* < 0.05), and LF (3.00 ± 1.12 mm vs. 5.89 ± 1.83 mm, *p* < 0.05).

**Table 1:** Patient profile and outcomes.

Case No.	Age (years)	Sex	Laterality of blepharoptosis	Total amount of LC resection (mm)	PFH (Preop/Postop) (mm)	MRD1 (Preop/Postop) (mm)	LF (Preop/Postop) (mm)	Functional outcome^a^	Aesthetic outcome^b^	Operation time (minutes)	Complications	Follow-up (Months)
1	4	F	Left	23	5/9	0/4	4/8	Excellent	Excellent	53	Transient lagophthalmos	12
2	5	F	Right	24	3/8	−2/3	2/5	Excellent	Excellent	50	Nil	6
3	8	M	Right	25	1/7	−4/2	4/6	Good	Excellent	60	Nil	9
4	6	M	Right	25	2/8	−3/3	3/4	Excellent	Excellent	50	Nil	10
5	12	M	Left	26	1/8	−4/3	2/3	Excellent	Excellent	52	Nil	8
6	3	M	Left	24	2/8	−3/3	2/5	Excellent	Excellent	55	Nil	7
7	6	F	Right	25	1/8	−4/3	3/6	Excellent	Excellent	53	Nil	5
8	7	F	Right	25	2/8	−3/4	5/8	Excellent	Excellent	50	Nil	8
9	5	F	Left	25	1/7	−4/2	2/5	Good	Excellent	55	Nil	3

Abbreviation: R, right; L, left; PFH, palpebral fissure height; MRD1, margin reflex distance 1; LC; levator complex; LF, levator function; N/A, not applicable.

a Functional outcomes were evaluated by postoperative MRD1 ≥+3 mm classified as excellent, MRD1 +2 mm as good, MRD1 +1 mm as fair, and MRD1 ≤0 mm as poor.

b Aesthetic outcomes were assessed by examining the difference in MRD1 (∆MRD1) between both eyes to evaluate eyelid symmetry. ∆MRD1 ≤ 1 mm were defined as excellent, 1–2 mm as good, and >2 mm as poor.^3^

**Figure 1:**
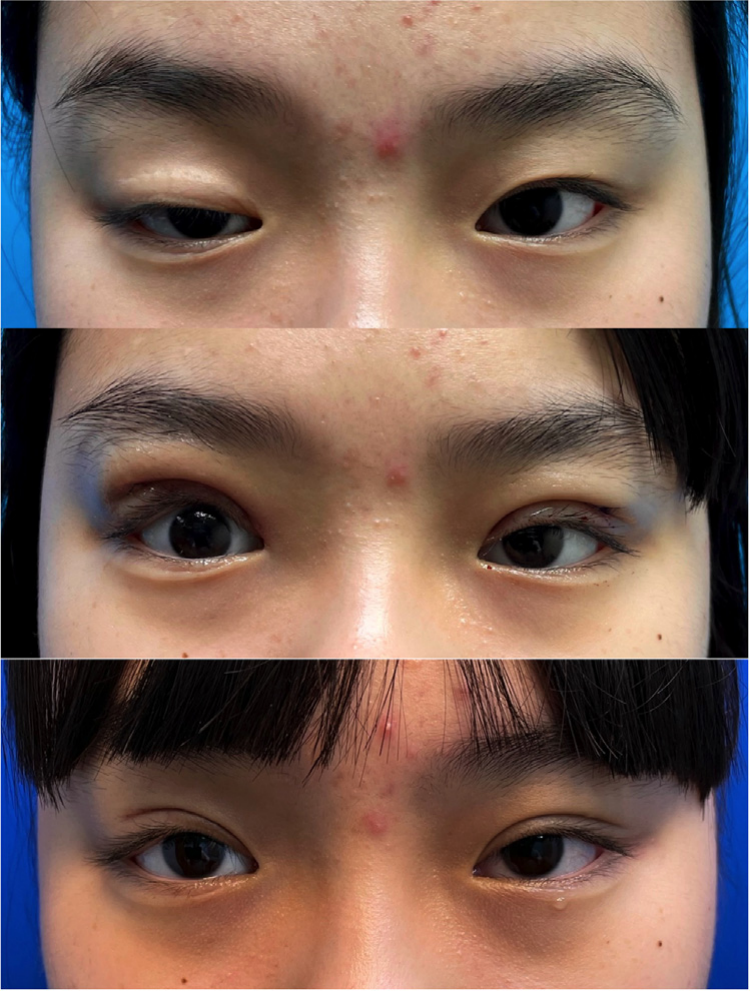
Preoperative image (top) of a 7-year-old girl with severe congenital blepharoptosis in her right upper eyelid. Preoperative PHF was 2 mm, MRD1 was −3 mm, and LF was 5 mm. One-month postoperative images (middle). Eight-month postoperative image (bottom) demonstrates a satisfying final outcome with. optimal function and symmetrical contour. Postoperative measurements included a PHF of 8 mm, MRD1 of 4 mm, and LF of 8 mm.

The mean follow-up period was 7.56 months. Functionally, seven of nine patients (77.78%) achieved excellent outcomes, and two patients demonstrated good functional outcomes. All patients (100%) exhibited excellent aesthetic results, with a ∆MRD1 ≤ 1 mm between both eyes. (Figure 1 and Table 1)

No cases of recurrent blepharoptosis were observed during follow-up. One patient experienced transient postoperative lagophthalmos, which resolved spontaneously within 1 month without intervention (SI 2).

## Discussion

HIAL technique offers a simplified and effective solution to these challenges by improving LF and minimizing the complication such as conjunctival prolapse, lagophthalmos, and eyelid retraction, particularly in pediatric patients. A thorough understanding of levator system anatomy is essential for performing the HIAL procedure. The upper eyelid levator system consists of three parallel arcs of fibrous tissue from lower to upper of the eyelid: the tarsus, the levator aponeurosis, and Whitnall’s ligament.^4^ The tarsal plate measures 6–10 mm in height from the upper eyelid margin.^4^^,^^5^ The distance between the levator muscle insertion and Whitnall’s ligament ranges from 14–20 mm. Beneath the levator aponeurosis, the conjunctiva adheres closely to muller’s muscle within 3–5 mm of the superior margin of the tarsal plate.^4^ Whitnall’s ligament provides a crucial support for the upper eyelid, lacrimal gland, and orbit by acting as a fixed fulcrum, redirecting the levator palpebrae superioris (LPS) from an anterioposterior to a vertical orientation during eyelid elevation. Additionally, the upper eyelid receives vascular supply from arterial arcades-marginal and peripheral arcade-located at distinct levels. The marginal arcade lies approximately 2–6 mm above the upper eyelid margin, whereas the peripheral arcade located about 9–11 mm, originates from the supratrochlear and supraorbital arteries.^4^ Recognizing these vascular structures is essential for minimizing intraoperative bleeding and avoiding injury.

Building on this anatomical foundation, the HIAL procedure was designed to optimize outcomes by dissecting the levator complex superiorly toward Whitnall’s ligament while preserving the upper eye lid’s vascular structure (SI 3).

## Conclusion

HIAL offers a streamlined approach for congenital ptosis with poor levator function, yielding favorable PFH and MRD1 outcomes while preserving anatomical integrity. Although LF improvements likely reflect enhanced mechanical efficiency, HIAL remains a viable surgical alternative. Future comparative studies are warranted to establish its long-term efficacy and clinical superiority before widespread adoption in the pediatric population.

## Funding

The authors received no financial support for the research, authorship, and/or publication of this article.

## Institutional review board statement

The study was conducted according to the guidelines of the Declaration of Helsinki and was approved by the Institutional Review Board of Kaohsiung Medical University Hospital [KMUHIRB-E(II)-20180282].

## Informed consent statement

Informed consent was obtained from patients’ legal guardians involved in the study.

## Reporting guideline statement

This study adhered to the STROBE (Strengthening the Reporting of Observational Studies in Epidemiology) guideline for observational studies.

## Data availability statement

The data presented in this study are available on request from the corresponding author. The data are not publicly available due to privacy and ethical concerns.

## Declaration of competing interest

The authors declare no conflicts of interest.

## Appendix Supplementary materials

Image, application 1

Image, video 2 dummy alt text
