# Supplementary material for: Refinement of maximal levator resection for blepharoptosis correction: High incision and advancement of levator complex
Source: JPRAS Open. 2026 Feb 10;49:133–8. doi: 10.1016/j.jpra.2026.02.004 (PMC12961211; doi:10.1016/j.jpra.2026.02.004)

**Supplementary Information**

SI 1. Schematic Illustration of HIAL Procedure.

(A) Dissection is meticulously performed to expose the levator complex (yellow. asterisk), extending upwards to Whitnall’s ligament (yellow arrow).

(B) A guidance from a horizontal marking situated 8 to 10 mm (upper marking) above. the upper tarsal border (lower marking).

(C) A rectangular flap, sized at 10 mm (vertical length) by 5 mm (horizontal length), is. Depicted. The vertical dimension of the flap is adjustable to achieve the desired positioning of the upper eyelid margin by the traction test.

(D) The flap is anchored to the midpoint of the tarsus using 6-0 nylon sutures (yellow. arrow heads). Precise alignment of the eyelid margin is achieved through meticulous medial and lateral sutures, aligning with the superior limbus border.


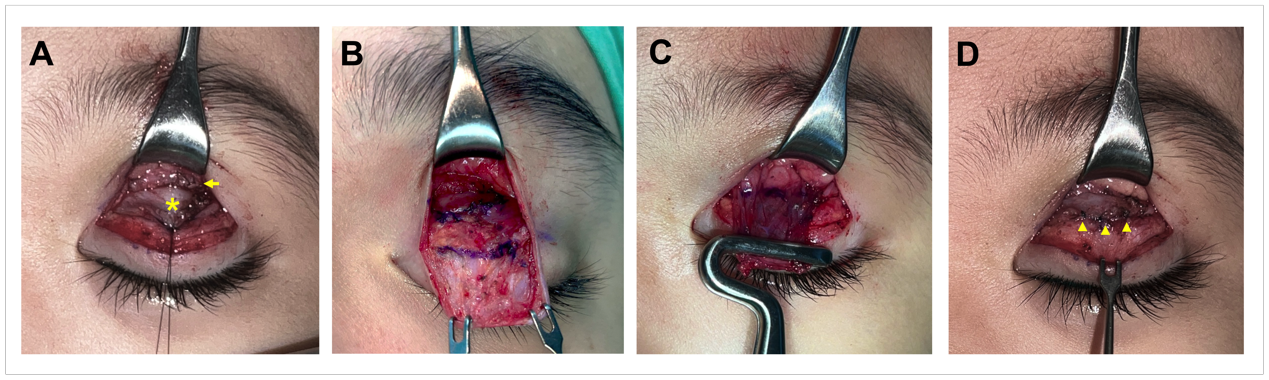


SI 2.

(A) Preoperative image of a 4-year-old girl with severe congenital blepharoptosis in her left upper eyelid. Preoperative PHF was 5 mm, MRD1 was 0 mm, and LF was 4mm.

(B) Immediate postoperative image showing lagophthalmos in left eye during attempted eyelid closure.

(C and D) One-month postoperative images. Eyes open (C) and eye closed (D). The. postoperative lagophthalmos resolved without intervention.

(E) Twelve-month postoperative image demonstrates a satisfying final outcome with optimal function and symmetrical contour. Postoperative measurements included a PHF of 9 mm, MRD1 of 4 mm, and LF of 8mm.


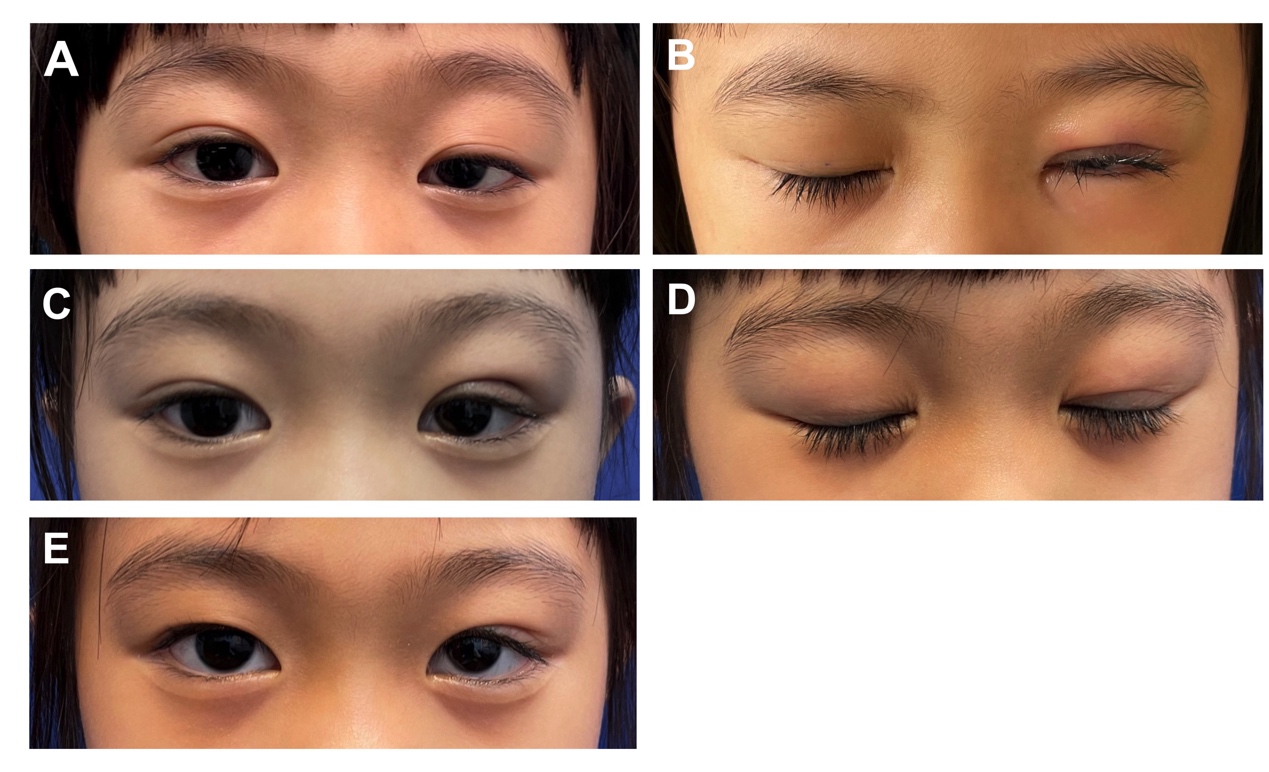


SI 3. Schematic comparison of HIAL versus MLR.

HIAL procedure (upper panel: A-C):

(A) A high incision is marked 8–10 mm above the superior tarsal border. Dissection proceeds superiorly along the levator complex toward Whitnall’s ligament, staying above the tarsus and away from the peripheral arcade.

(B) The levator complex flap is separated from the underlying conjunctiva and. advanced to the tarsus, preserving the suspensory support of the upper palpebral conjunctiva.

(C) The flap is anchored to the midpoint and upper third of the tarsus with 6-0 nylon. Eyelid height is refined by aligning with the superior limbus and adding medial and lateral sutures. Conjunctival prolapse is avoided because conjunctival support is preserved.

Mechanism of levator resection-associated complications (lower panel: D-F):

(D) An incision is made relatively close to the peripheral arcade, increasing the risk of vascular injury.

(E) The redundant length of levator complex is measured and resected, which disrupts conjunctival suspensory support.

(F) The anterior surface of the residual levator complex is anchored to the tarsus with 6-0 nylon; however, the loss of conjunctival support predisposes to conjunctival prolapse.


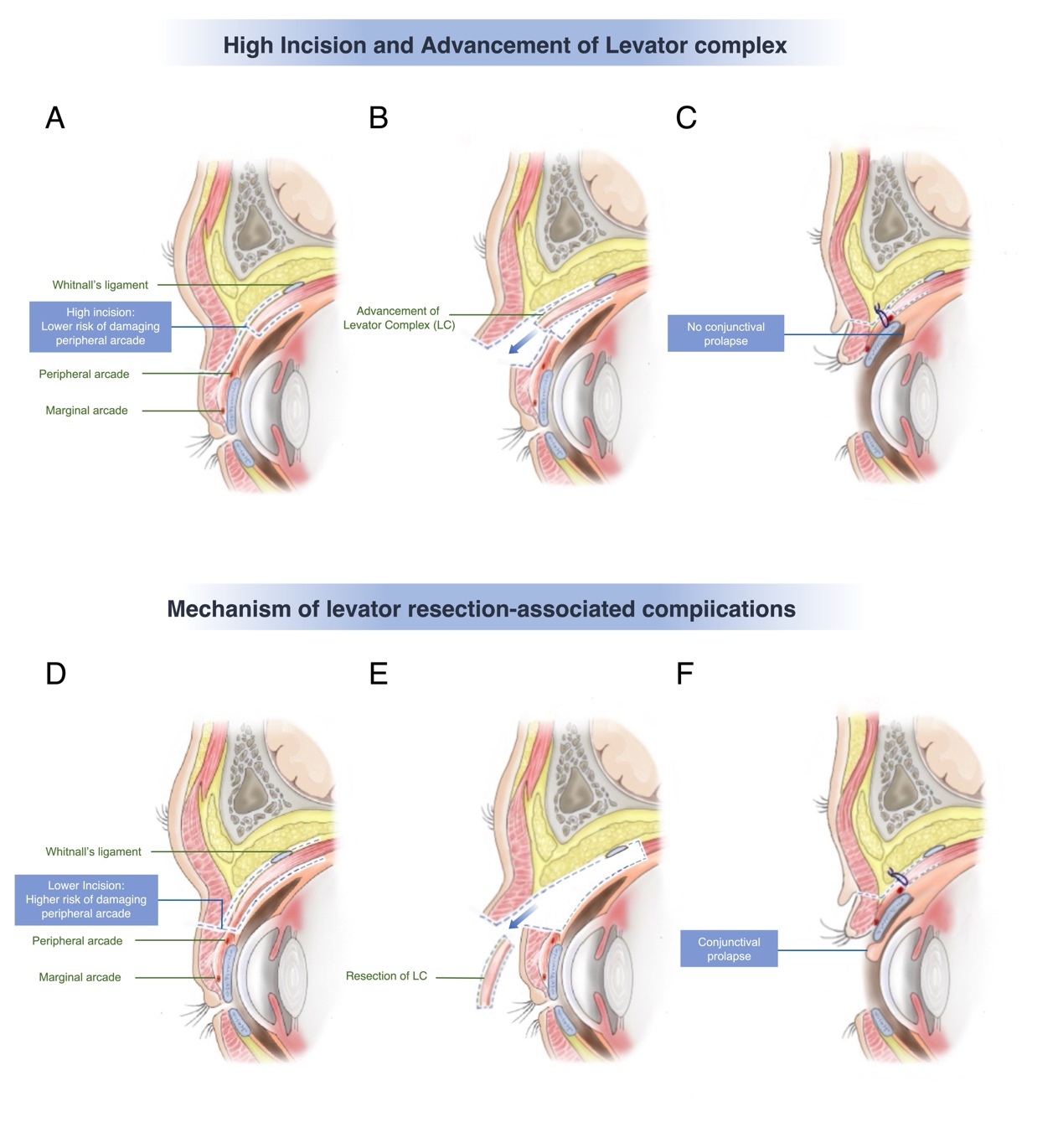

Supplement: Supplementary file 1 [file mmc1.docx]
